# Natural deep eutectic solvents as a green extraction of polyphenols from spent coffee ground with enhanced bioactivities

**DOI:** 10.3389/fpls.2022.1072592

**Published:** 2023-01-11

**Authors:** Aitor García-Roldán, Léa Piriou, Paula Jauregi

**Affiliations:** ^1^ AZTI member of, Food Research, Basque Research and Technology Alliance (BRTA), Derio-Bizkaia, Spain; ^2^ Polytech’Lille - Génie Biologique et Alimentaire, Université de Lille, Villeneuve d’Ascq, France; ^3^ Ikerbasque, Basque Foundation for Science, Bilbao, Spain

**Keywords:** polyphenols, antimicrobial activity, antioxidant activity, natural deep eutectic solvent (NADES), selectivity, extraction, spent coffee ground

## Abstract

Conventional extraction techniques are usually based on highly pollutant and/or flammable organic solvents. Therefore, alternative environmentally friendly extraction methods are of particular interest for the recovery of bioactive compounds for their application as food ingredients and/or nutraceuticals. Natural deep eutectic solvents (NADES) are a green and nontoxic attractive alternative to hydroalcoholic extraction. NADES media primarily depends on the intermolecular interactions (hydrogen bonding) among their components to form a eutectic mixture with a much lower final melting point than its individual components. Examples of natural deep eutectic NADES solvents include aqueous solutions (25%–50% water) of choline chloride, sugars, and polyols. This study aimed to investigate the application of two NADES, namely, betaine:triethylene glycol (Bet : TEG) and choline chloride:1,2-propanediol (Chol : Prop), as sustainable green solvents for the extraction of polyphenols from spent coffee ground (SCG), a by-product of coffee processing. In particular, the extraction yield and selectivity were evaluated and compared with conventional green extractions (hot water and a hydroalcoholic solution). In addition, the effect of NADES on the antioxidant and antimicrobial activity of the extracts was investigated. The main outcomes were as follows: (i) NADES were as effective as other conventional green solvents in the extraction of polyphenols with the added advantage of operating at milder temperature conditions, without flammable solvents and with sustainable and natural compounds; (ii) the antimicrobial activity of the NADES extracts was 10 times higher than that of the ethanolic and aqueous extracts. Given the low toxicity of NADES, they could be used as formulation aid for food ingredients.

## 1 Introduction

Bioactive extraction from plant biomass is one method for agro-industrial by-product valorization. Conventional extraction techniques are usually based on highly pollutant and/or flammable organic solvents (Okur et al., 2020; Pettinato et al., 2020), frequently assisted by other technologies, such as ultrasound-assisted extraction (UAE) (Soria and Villamiel, 2010) or high hydrostatic pressure-assisted extraction (HHPE) (Okur et al., 2020). Therefore, alternative environmentally friendly extraction methods are of particular interest for the recovery of bioactive compounds for their application as food ingredients and/or nutraceuticals. Natural deep eutectic solvents (NADES) emerge as an alternative ecofriendly extraction method. Deep eutectic solvents are composed of a hydrogen bond donor (HBD) and hydrogen bond acceptor (HBA), creating strong hydrogen bonds between them (Cui et al., 2018) and resulting in a eutectic mixture with a drastic reduction of the melting point, which is more suitable for thermolabile compound extraction (Oomen et al., 2020; Fuad et al., 2021). The high viscosity of NADES can hinder their extractive efficiency, which can be reduced by adding a certain percentage of water or by working at higher temperatures. However, high dilutions with water can result in a disruption of the supramolecular structure of NADES as water molecules will compete with NADES components for hydrogen bonding. Hence, at a water content higher than 50%, NADES may be considered an aqueous solution to its constituents rather than a eutectic mixture with the subsequent changes in physicochemical properties and possibly solubility capacity (Dai et al., 2015). This supramolecular structure gives NADES the ability to partially disrupt plant cell wall by the formation of hydrogen bonds between cell wall constituents and the eutectic mixture (Zdanowicz et al., 2018; Chen and Mu, 2019; Liu et al., 2019). Typically, NADES components are sugars, amino acids, and organic acids, which are inexpensive, easy to synthesize and/or of natural origin, biodegradable, and nontoxic; therefore, they are an interesting alternative to organic solvents (Cui et al., 2018; Alibade et al., 2021; Fuad et al., 2021). Even more, NADES solvents’ GRAS (generally recognized as safe) status makes them appropriate for food applications (Fuad et al., 2021). Furthermore, NADES have been reported to have beneficial effects on the bioavailability of the extracted bioactives (Da Silva et al., 2020; Tong et al., 2021), and recently, Wojeicchowski et al. (2021) found that propylene glycol and choline chloride-based NADES contributed to the antimicrobial activity of the extracts against both Gram-positive and Gram-negative bacteria.

Spent coffee ground (SCG) is the main by-product of coffee processing, which is principally produced in coffee shops and restaurants, reaching a production of several tons per year worldwide (Jiménez-Zamora et al., 2014 Hudecvoka et al., 2018). Among SCG’s components, polyphenols, caffeine, and melanoidins have shown many benefits for human health, making it a valuable source of bioactive compounds with proven antioxidant, antibacterial, antiviral, anti-inflammatory, anticarcinogenic, and prebiotic effects (Jiménez-Zamora et al., 2014; Yoo et al., 2018; Pettinato et al., 2020). Bioactive compound extraction from SCGs has been widely studied, and more recently, NADES have also been successfully used in the extraction of these functional metabolites (Yoo et al., 2018; Zdanowicz et al., 2018; Oomen et al., 2020; Ahmad et al., 2021). Fanali et al. (2020) tested several choline chloride and betaine-based NADES and found that betaine:triethylene glycol was the most effective in polyphenol extraction from SCG (Fanali et al., 2020), but selectivity of the extraction was not evaluated. Both choline and betaine contain a positively charged amine group, which could promote electrostatic interactions with negatively charged/polarized polyphenols. Previously, authors found that extraction of polyphenols from grape marc was enhanced when using a cationic surfactant as compared with a nonionic surfactant, which was ascribed at electrostatic interactions with the former (Spigno et al., 2015).

This study aimed to investigate the application of two NADES, namely, betaine:triethylene glycol (Bet : Teg) and choline chloride:1,2-propanediol (Chol : Prop), as sustainable green solvents for the extraction of polyphenols from SCG. In particular, the extraction yield and selectivity were evaluated and compared with conventional green extractions (specifically hot water and an ethanolic aqueous solution). In addition, the effect of NADES on the antioxidant and antimicrobial activity of the extracts was investigated.

## 2 Materials and methods

### 2.1 Chemicals

The methanol and acetonitrile used were HPLC (high-performance liquid chromatography) grade and purchased from Fisher Scientific, as well as the potassium sodium tartrate tetrahydrate used for reducing sugar determination and D-glucose standard (Madrid, Spain). Choline chloride, betaine, 1,2-propanediol, and albumin standard were from Thermo Fisher Scientific (Madrid, Spain), and triethylene glycol and Trolox standard were supplied by Acros Organics (Antwerp, Belgium). The HPLC standards, trifluoroacetic acid, and 2,2-diphenyl-1-picrylhydrazyl (DPPH) were purchased from Sigma Aldrich (St. Louis, MO, USA)l; Folin-Ciocalteu reagent and ethanol were obtained from Scharlau (Barcelona, Spain); and 3,5-dinitrosalicylic acid was purchased from Alfa Aesar (Kandel, Germany). Mueller–Hinton Broth culture media and bacteriological agar for microbiological assays were supplied by Oxoid (Basingstoke, Hampshire, UK).

### 2.2 Samples

SCG samples were provided by Euskovazza S.L. (Usurbil, Gipuzkoa, Spain), recovered from express coffee brewing machines, and they were composed of Robusta and Arabica varieties. After arrival, samples were dried at 100°C for 48 h in a forced-air oven (SELECTA, Barcelona, Spain), until a water content lower than 1% was reached. Dried samples were then stored in thermically sailed plastic bags at 40°C for further analysis. The typical composition of the SCG was 62.28% carbohydrates in dry weight (DW), cellulose and hemicellulose being the main ones, 12.49% protein, 16.36% fat, and 1.48% ashes (San Martín et al., 2020).

### 2.3 Aqueous extraction

Aqueous extraction was carried out at a sample:solvent ratio of 1:8 (g DW:ml) at 100°C in a magnetic agitation-assisted water bath for 1 h. These conditions were chosen based on those found optimum in a previous work by the authors on the extraction of polyphenols from grape marc (Mohd Maidin et al., 2018). The solid and liquid phases were separated by centrifugation at 10,000×*g* for 10 min and the supernatant was filtered through a 45-µm pore size filter. Each extraction was carried out in triplicate for appropriate statistical representation.

### 2.4 Ethanolic extraction

An aqueous ethanolic solution (60% ethanol) was used in a sample:solvent ratio of 1:8 (g DW:ml), at 60°C in a water bath with magnetic agitation for 2 h. These conditions were chosen based on those found optimum in a previous work by the authors on the extraction of polyphenols from grape marc (Mohd Maidin et al., 2018). The solid and the liquid phases were separated by centrifugation at 10,000×*g* for 10 min, and the supernatant was filtered through a 45-µm pore size filter. Each extraction was carried out in triplicate for appropriate statistical representation.

### 2.5 NADES synthesis and extraction

Betaine:triethylene glycol NADES (Bet : Teg) synthesis and extraction were performed as described by Fanali et al. (2020) with some modifications. The solvent was prepared by mixing betaine and triethylene glycol at a molar ratio of 1:2 for 30 min at 80°C and adding 30% or 40% of deionized water (Bet : Teg 70% and 60%, respectively). The mixture was stirred by magnetic agitation until a colorless solution was obtained. The extraction was carried out at a sample:solvent ratio of 1:15 (g DW:ml) and 65°C in a magnetic agitation-assisted water bath. The solid and liquid phases were separated by centrifugation at 10,000×*g* for 10 min and the supernatant was filtered through a 45-µm pore size filter.

Choline chloride:1,2-propanediol NADES (Chol : Prop) preparation was done as described by Wojeicchowski et al. (2021), briefly by mixing both components at a 1:2 molar ratio in a magnetic stirrer at 70°C until a colorless solution was obtained. Then, 40% and 50% of deionized water was added (Chol : Prop 60% and 50%, respectively). The sample:solvent ratio was set at 1:15 (g DW:ml), and the extraction was carried out at 65°C in a water bath equipped with a mechanical agitation system for 150 min. The separation of solid and liquid phases was done as explained before. Each extraction was carried out in duplicate.

### 2.6 Viscosity determination of NADES at varying water composition

The characterization of the NADES was done by measuring their viscosity at different dilutions ranging from 15% to 70% of water, with a Brookfield DV-E Viscometer (Brookfield, Middleborough, MA, USA), using the S62 spindle at 60 rpm. Measurements were done in duplicate and CV < 5%.

### 2.7 Total phenolic content assay

The total phenolic content was measured by Folin-Ciocalteu colorimetric assay, adapted for a 96-microwell plate spectrophotometer (Thermo Fisher Scientific, Roskilde, Denmark). Briefly, 140 µl of each sample or gallic acid standard reacts with 30 µl of Folin-Ciocalteu reagent and 140 µl of 7% Na_2_CO_3_ solution, and absorbance was measured at a wavelength of 750 nm after 1 h of dark incubation. Measurements were carried out in triplicate with a coefficient of variance (CV%) lower than 5%. Quantification was based on calibration curves of gallic acid standard where the equation was *y* = 0.0473*x* + 0.0726 (*R* ^2^ = 0.9993). The results were expressed as gallic acid equivalents (GAE) (Munteanu and Apetrei, 2021). Samples from Chol : Prop extracts were diluted 500-fold to avoid precipitation as it was observed that as soon as the sample was put in contact with the Folin reagent, the sample became turbid and a fine precipitate appeared, which can affect the spectrophotometric assay.

### 2.8 Antioxidant activity

The DPPH radical scavenging activity (DRSA) method was used for antioxidant activity determination (Brand-Williams et al., 1995) with slight modifications. Briefly, a 25 ppm DPPH (2,2-diphenyl-1-picrylhydrazyl) solution in methanol was prepared, and 280 µl was added to 20 µl of sample or Trolox standard in a 96-microwell plate. After incubating for 30 min at room temperature in darkness, absorbance was measured at λ = 515 nm. Measurements were carried out in triplicate and coefficient of variability was lower than 5%. Quantification was based on Trolox standard calibration curves where the equation was *y* = −0.0049*x* + 0,8621 (*R* ^2^ = 0,9981). Results were reported as Trolox equivalent antioxidant capacity (TEAC) in µmol of Trolox equivalents per gram of dry matter.

### 2.9 Total protein content

Total protein content was measured by a bicinchoninic acid assay (BCA) commercial kit (Thermo Fisher Scientific, Roskilde, Denmark). As the protocol explains, 10 µl of the sample was mixed with 200 µl of the reagent in a 96-microwell plate and incubated for 30 min at 37°C. Calibration curve was constructed with albumin. To avoid sugar–reagent interactions, which can lead to erroneous measurements, protein precipitation with acetone was carried out as explained in the protocol. The spectrophotometer was set at λ = 550 nm for protein quantification. Measurements were carried out in triplicate and coefficient of variability was lower than 5%. Quantification was carried out based on an albumin standard calibration curve where the equation was *y* = 0.5241*x* + 0.0653 (*R* ^2^ = 0.9979). Results were reported as % of protein in dry matter.

### 2.10 Total reducing sugar content

Dinitrosalicylic (DNS) acid assay was used for total reducing sugar content determination, adjusted to a 96-well microplate. The DNS reagent preparation was done by dissolving 8 g of NaOH in 100 ml of distilled water and adding 5 g of DNS, 250 ml of distilled water, and 150 g of potassium sodium tartrate tetrahydrate. The assay was carried out by placing 25 µl of blank, standard of D-glucose or sample, and 25 µl of DNS reagent in each well. The microplate was incubated for 10 min at 100°C and cooled down in an ice bath before diluting it and measuring absorbance at 540 nm. Measurements were carried out in triplicate and coefficient of variability was lower than 5%. Quantification was done by constructing a glucose standard calibration curve where the equation was *y* = 0.4093*x* + 0.0295 (*R* ^2^ = 0.9934).

### 2.11 HPLC-UV/Vis qualitative analysis

Chromatographic assays were performed following the method reported by Navarra et al. (2017) with an Agilent Technologies 1200 series HPLC system (Santa Clara, CA, USA) equipped with a UV/Vis photodiode array detector, a quaternary pump and a degasser system. The column used was Phenomenex Gemini 5u C18 110A (Phenomenex, Torrance, CA, USA) at room temperature. The mobile phases used were trifluoroacetic acid 0.1% in water (solvent A) and acetonitrile (solvent B), which were pumped at 1.5 ml/min in the following gradient: 95% of A at time 0 min; 80% of A at time 20 min; 80% of A at time 30 min; and 95% of A at time 35 min. Twenty microliters of each sample and standard was injected and absorbance was measured at 272 and 326 nm of wavelength.

The standards used were caffeine, caffeic acid, gallic acid, and 3-O-caffeoylquinic acid, and the absorption spectrum of each molecule was recorded from 200 to 400 nm for further identification in samples. The calibration curves were constructed ranging from 0.5 to 200 mg/L in each solvent and reaching up to 500 mg/L for caffeine.

### 2.12 Antimicrobial activity

Foodborne pathogen and spoilage bacteria strains were chosen for the antimicrobial activity tests. Antimicrobial activity was first tested by agar diffusion method as explained elsewhere (Ibarruri, 2019) with some modifications. The target microorganisms *Staphylococcus aureus* (CECT 435), *Salmonella enterica* (CECT 4156), *Bacillus subtilis* (CECT 13), *Bacillus cereus* (CECT 131), and *Escherichia coli* (CECT 516) were cultivated in Mueller–Hinton (MH) agar plates at 37°C for 24 h and then transferred to 10 ml of MH broth tubes at an optical density of 0.50 (approximately 10^8^ cfu/ml). Two hundred microliters of culture was spiked in 8 ml of MH soft agar, vortexed, and poured into MH agar plates. Once dried, 10 µl of each sample was added, and after 24 h of incubation at 37°C, the diameters of zone inhibition were measured.

The samples that showed zone inhibition were tested for minimum inhibitory concentration (MIC). Different dilutions of each sample were prepared in MH broth and filtered through 0.45-µm pore size PVDF sterile filters. Target microorganisms were spiked in MH broth tubes at an optical density of 0.10 for *B. cereus* and *E. coli* and 0.05 for *B. subtilis* and diluted at a 1:100 ratio (*B. cereus* and *B. subtilis*) or 1:1,000 ratio (*E. coli*). One hundred microliters of each sample and 100 µl of culture were added to 100-microwell sterile plates and optical density was measured at λ = 600 nm every 30 min for 24 h at 37°C by the BioScreen C Automated Microbiology Growth Curve Analysis System (Labsystems, Helsinki, Finland). The MIC was determined as the average of triplicate growth curves for each microorganism.

### 2.13 Statistical analysis

Statistical analyses were carried out by the software Statgraphics Centurion XVI 16.2.04 (Statgraphics Technologies Inc., Virginia, USA). Normality of the data was checked by Shapiro–Wilk test. One-way ANOVA was applied to test the significance of difference in the yield of extraction of polyphenols, protein, and sugar and antioxidant activity between the different solvents including the different water compositions. Kruskal–Wallis test was used for nonparametric data. Student’s *t*-test was applied to compare the microbiological inhibitory effect of the extract with the solvent alone. Significant differences were claimed for all analysis at *p*-value < 0.05.

## 3 Results and discussion

### 3.1 NADES extraction conditions

**Figure 1:**
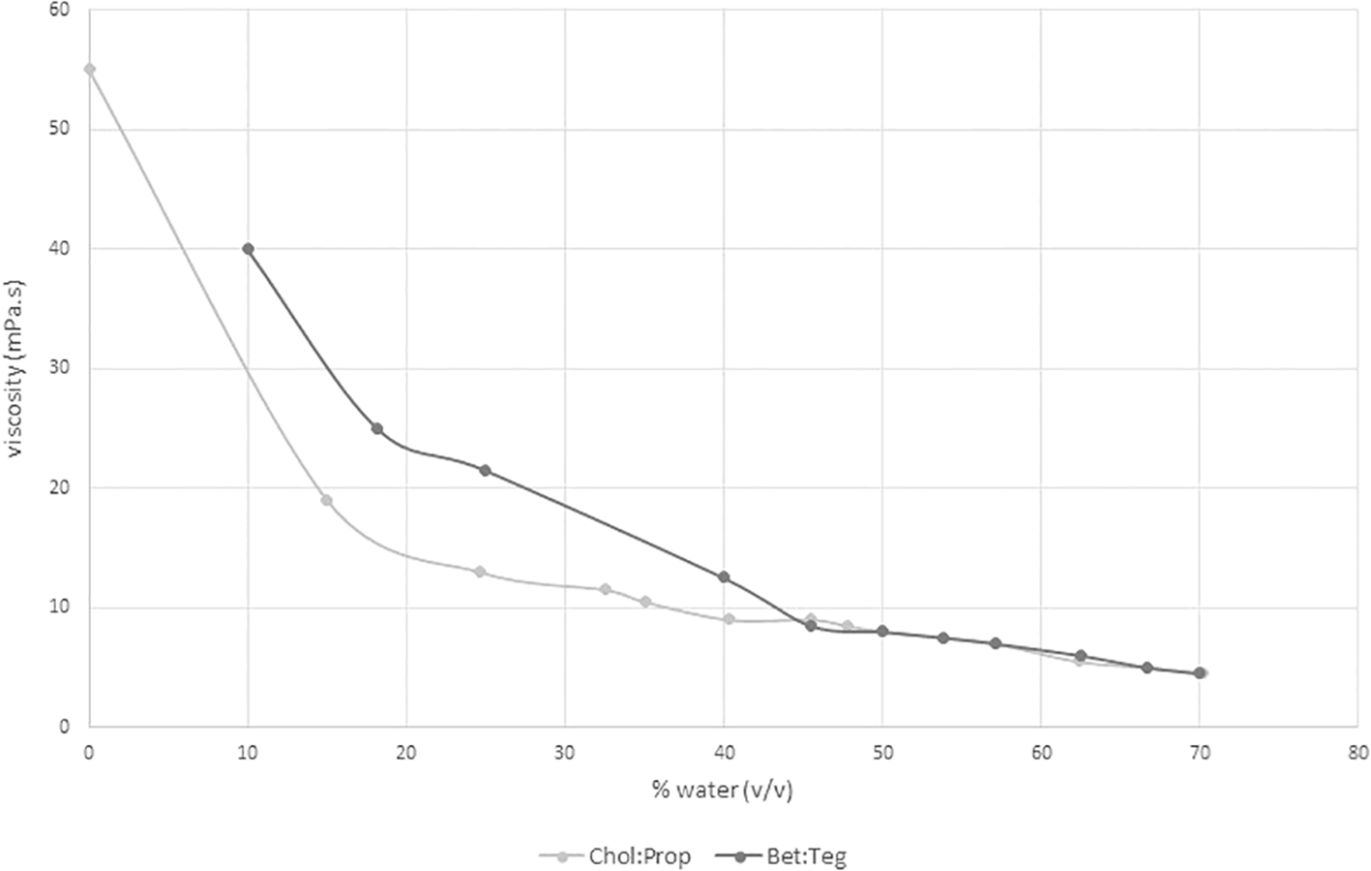
Viscosity evolution of both NADES with different dilution factors.

Extraction conditions were based on a recently published work (Fanali et al., 2020). Water composition will have an effect on viscosity of NADES by reducing it and this, in turn, can increase extraction efficiency by improving mass transfer. Here, the following water compositions were chosen: 30% and 40% corresponding to 70% and 60% Bet : Teg, and 40% and 50% corresponding to 60% and 50% Chol : Prop. The effect of water composition on viscosity is shown in **Figure 1**. In the case of Chol : Prop, the chosen water compositions fell within the plateau area as, after 35% water, there was no significant change in viscosity (8–9 mPa·s). This change in viscosity may be a reflection of changes in the supermolecular structure of NADES; Dai et al. (2015) reported that Chol : Prop diluted with more than 50% water led to complete disruption of the hydrogen bonding between its components and resulted in aqueous solution of its components. On the other hand, Bet : Teg had a higher viscosity than Chol : Prop, and at the water compositions, the chosen viscosity had not reached a plateau (about 20 and 12.5 mPa·s at 30% and 40% water, respectively).

The extraction method was based on Fanali´s where they used UAE; however, when UAE was tested against mechanical agitation, no statistically significant differences were found between both methods (data not shown). Therefore, subsequent extractions were carried out with mechanical agitation. Furthermore, extraction time was selected based on the extraction kinetics obtained for each of the NADES at the different water compositions tested (data not shown). Polyphenol yield reached equilibrium at 45 min for Bet : Teg and at 150 min for Chol : Prop. Based on this, the subsequent extractions were carried out at these equilibrium times.

### 3.2 Extraction efficiency comparison

**Figure 2:**
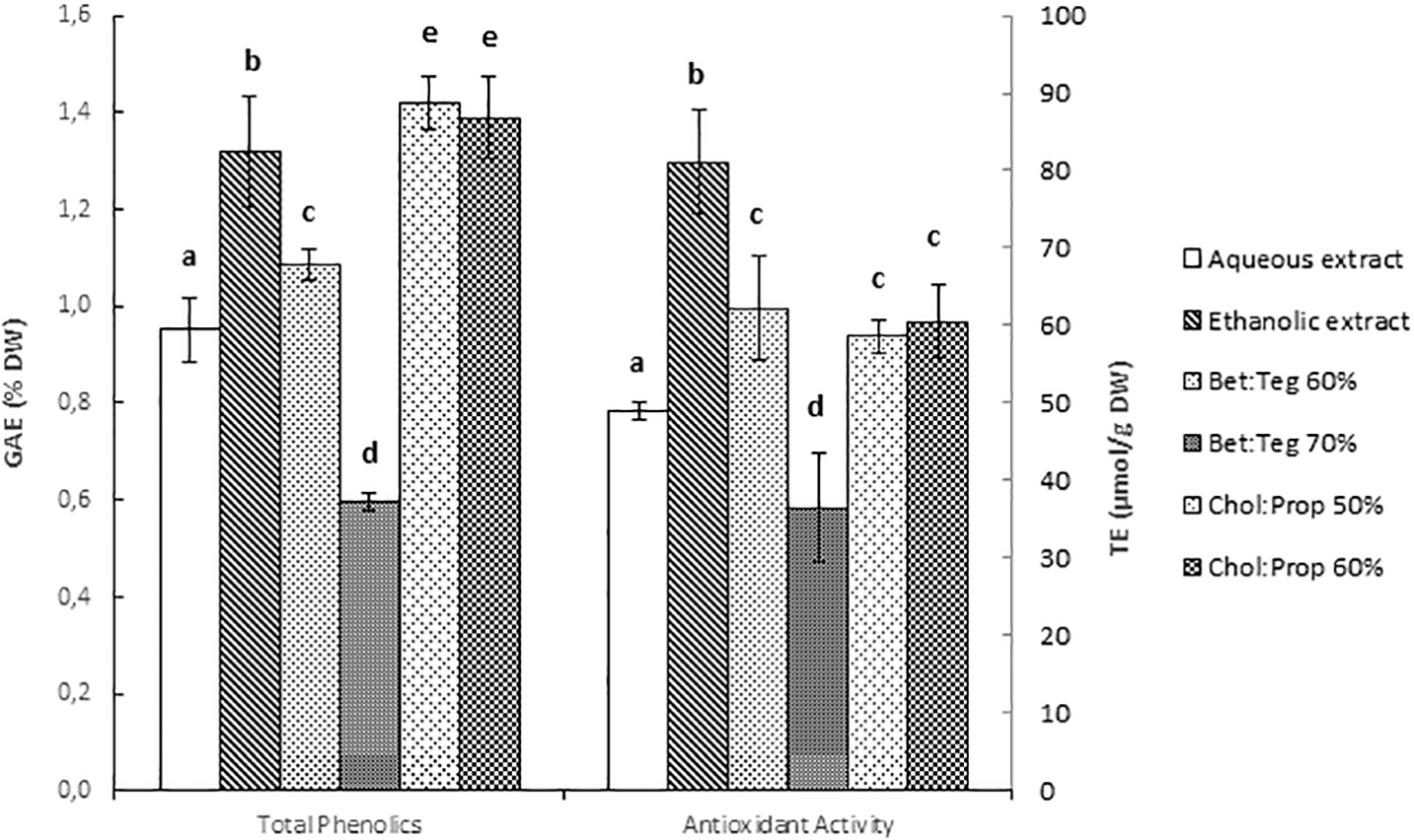
Total phenolics (TP) content expressed in gallic acid equivalents (GAE) % of dry weight (DW) and antioxidant activity expressed in Trolox equivalent (TE) µmol/g DW. Letters above error bars stand for statistically significant differences between groups.

The extraction efficiency of the different solvents for polyphenols was evaluated. The best extraction yield was obtained with Chol : Prop (1.4% DW), even slightly higher than with ethanol (1.3% DW), and no significant differences were seen between 50% and 60% Chol : Prop (**Figure 2**). Results obtained here were within those previously reported for SCG with other solvents. For example, Mussatto et al. (2011) extracted 1.6% (DW) polyphenols with 60% MeOH (90-min extraction and a solvent:sample ratio of 40 ml/g) (Mussatto et al., 2011). Ballesteros et al. extracted up to 4% total polyphenols but under harsher conditions with water, at 200°C and 50 min (Ballesteros et al., 2017).

The antioxidant activity followed the same trend as the total polyphenol content for all the solvents, indicating that the phenolics were the main compounds contributing to the antioxidant activity, except for Chol : Prop. These extracts showed slightly lower activity than the ethanolic extract, which could be due to differences in polyphenol profile or due to other non-polyphenols that were not determined (e.g., melanoidins) but contributed to the antioxidant activity.

One of the main polyphenols in coffee and SCG is the 3-O-caffeoylquinic acid, which is part of the chlorogenic acids (CGA). This was determined together with caffeic acid. Overall, the same trend as for total polyphenols was observed, and the values agreed with those reported by Okur et al. (2020). The highest concentrations of these three polyphenols were found in the water extract (**Table 1**) despite the total polyphenols being higher in the ethanol extract (**Figure 2**). As gallic acid, 3-O-caffeoylquinic acid, and caffeic acid are polar polyphenols, they are more soluble in water, reaching a higher extraction yield (60%) than with ethanol. Similarly, Bet : Teg and Chol : Prop at 60% had a higher content of these polyphenols, showing that the extraction capacity was higher for NADES than for ethanol. The highest concentration of CGA was found in the water extract but followed closely by Chol : Prop at 60%. Despite this, the ethanolic extract had greater content of total phenolics and antioxidant activity, which means that other antioxidant compounds are being extracted.

**Table 1:** Gallic acid, 3-O-caffeoylquinic acid (CGA), and caffeic acid content (average values of three replicas) expressed in mg/kg of SCG.

Sample	Gallic acid	CGA	Caffeic acid
Aqueous extract	99.09 ± 0.93	142.74 ± 7.52	53.80 ± 1.92
Ethanolic extract	83.45 ± 15.90	89.33 ± 3.66	37.14 ± 2.09
Bet : Teg 60%	154.73 ± 0.82	116.09 ± 18.08	59.64 ± 0.84
Bet : Teg 70%	78.16 ± 15.95	71.70 ± 10.48	40.28 ± 0.07
Chol : Prop 50%	138.5 ± 1.09	131.34 5 ± 0.75	63.17 ± 0.53
Chol : Prop 60%	126.83 ± 1.47	128.18 ± 2.18	59.92 ± 0.24

Fanali and coworkers found that a higher yield of CGA (based on total CGA determination) was obtained with betaine-based NADES (0.46%) instead of choline (0.28%) (Fanali et al., 2020), contrary to what was found here. Moreover, they claimed that 70% Bet : Teg performed better than 60%, whereas here, 60% had better results in terms of extraction yield. This could be partly due to a viscosity effect as, at higher water content, the reduced viscosity (**Figure 1**) would improve mass transfer. Also, the more polar environment could favor SCG phenolics extraction. On the other hand, Chol : Prop did not show significant differences between 50% and 60% dilution factors, which would confirm the viscosity effect as there were no changes in viscosity in this region (**Figure 1**).

In summary, Chol : Prop showed better performance in terms of polyphenol extraction yield than Bet : Teg with 1.4% DW and 1.1% DW, respectively, as well as in terms of CGA and caffeic acid yields. The differences in extraction efficiency might be due to the differences in interaction between polyphenols and NADES components. Choline has a positively charged quaternary amine group, which could promote electrostatic interactions with the negatively charged/polarized polyphenols and, in this way, enhanced their extraction. On the other hand, betaine has both a positively charged amine and a negatively charged carboxylic group at the pH of the extract (pH >5.5), which can hinder electrostatic attractive interactions with polyphenols. The higher viscosity of Bet : Teg than Chol : Prop could also have contributed to the lower yield of extraction. However, viscosity could have little effect when comparing Bet : Teg 60% with Chol : Prop as these systems had similar viscosities (**Figure 1**).

### 3.3 Extractant selectivity for different bioactive compounds

**Figure 3:**
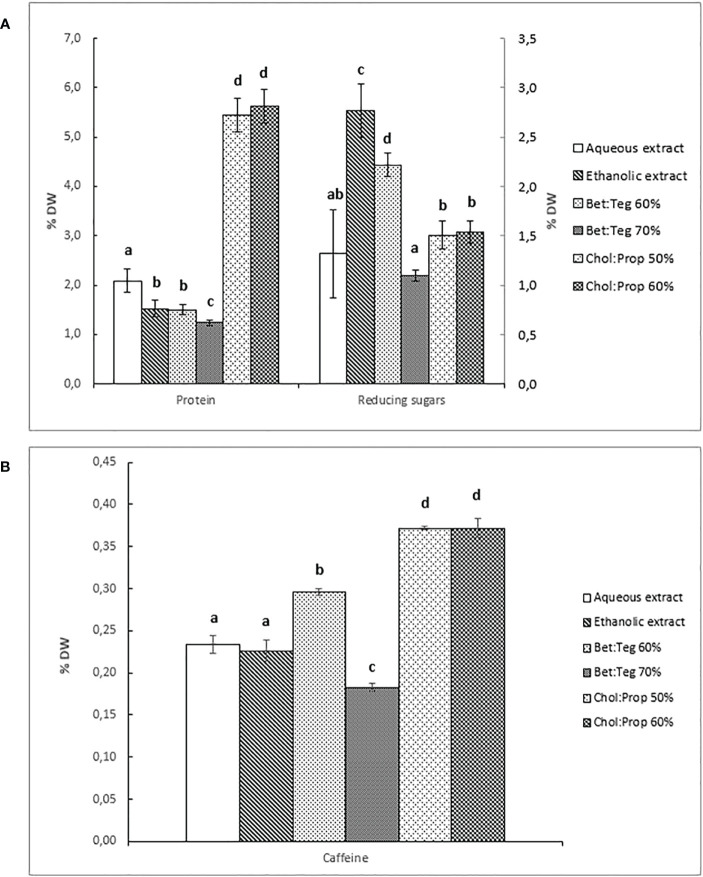
**(A)** Protein and reducing sugars content expressed in % of dry weight (DW). **(B)** Caffeine content expressed in % of dry weight (DW). Letters above error bars stand for statistically significant differences between groups.

As an important aspect of the evaluation of the extraction capacity of each solvent, the selectivity of the extraction in relation to other key components, such as protein, sugar, and caffeine, was evaluated. Each extractant showed different selectivity for the analyzed compounds. Chol : Prop extracts showed by far the highest selectivity for proteins (**Figure 3A**) and caffeine (**Figure 3B**), while ethanol was the most selective for reducing sugars followed closely by Bet : Teg (60%) (**Figure 3A**). Following the same trend observed before for polyphenols (**Figure 2**), Bet : Teg showed better performance at 60% than 70% for protein, reducing sugars and caffeine.

**Figure 4:**
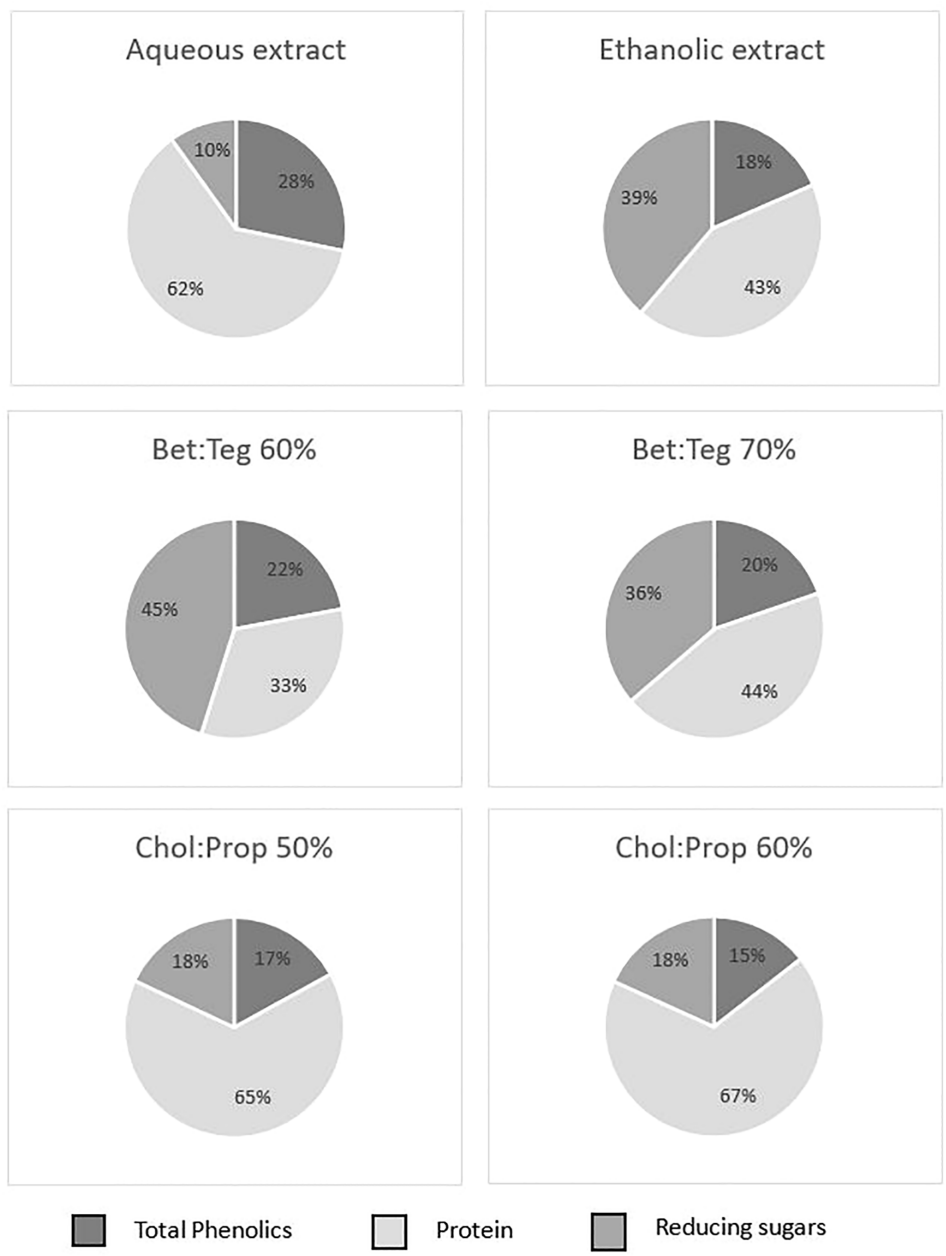
Pie charts representing the composition of phenolics, protein, and reducing sugars for each extract.

In order to better visualize the differences in selectivity of the extractants, which, in turn, resulted in different extract compositions, pie charts with the main components, namely, polyphenols, protein, and reducing sugars, are shown in **Figure 4** where the total (100%) is the sum of three components. These clearly show the higher selectivity of Chol : Prop for proteins and of ethanol for sugars. Moreover, it is interesting to highlight that in both water and Chol : Prop extracts, protein proportion is much higher (62%–67%) than reducing sugars (10% and 18%), while in both ethanol and Bet : Teg extracts, the protein is lower (33%–44%) and reducing sugar is higher (36%–45%). It could be hypothesized that protein extraction is favored over sugars in Chol : Prop due to the charge effect while Bet : TEG favored the extraction of sugars over protein.

Thus, the aqueous and Chol : Prop extracts with high polyphenol and protein content but low sugar content have a more favorable composition for their potential application as a food ingredient than the other extracts.

### 3.4 Antimicrobial activity and minimum inhibitory concentration

Among the five tested microorganisms, the ethanolic and aqueous extracts showed clear zone inhibition for *B. subtilis* and *B. cereus*, and slight inhibition for *E. coli.* Meanwhile, Bet : Teg extracts only showed slight inhibition for *B. subtilis* and *B. cereus*, and Chol : Prop extracts for *E. coli* and *B. cereus*. These microorganisms were selected for the determination of the minimum inhibitory concentration (MIC) of all extracts.

The MIC of total phenolic compounds for each extract represented in mg/L is shown in **Table 2**. Aqueous and ethanolic extract had a 10-fold greater MIC than NADES extracts, which were approximately 3,000 mg/L and below 300 mg/L, respectively. Further assays were carried out with the NADES solvents alone, and it was found that they had inhibitory activity for all bacteria, even at concentrations as low as 15% (v/v) for *B. cereus*, *B. subtilis*, and *E. coli* and 30% (v/v) for *B. subtilis* in the case of Chol : Prop. Therefore, the inhibitory effect cannot be attributed only to the phenolics extracted by NADES solvents.

**Table 2:** Minimum inhibitory concentration in mg/L of total phenolics.

Sample	*E. coli*	*B. subtilis*	*B. cereus*
Aqueous extract	3,000 (25)	>3,000 (25)	3,000 (25)
Ethanolic extract	3,000 (25)	1,500 (12.5)	1,500 (12.5)
Bet : Teg 60%	300	150	300
Bet : Teg 70%	200	100	200
Chol : Prop 50%	300	300	300
Chol : Prop 60%	300	300	300

The values in brackets for aqueous and ethanolic extract show the total solid concentration in mg/ml.

**Figure 5:**
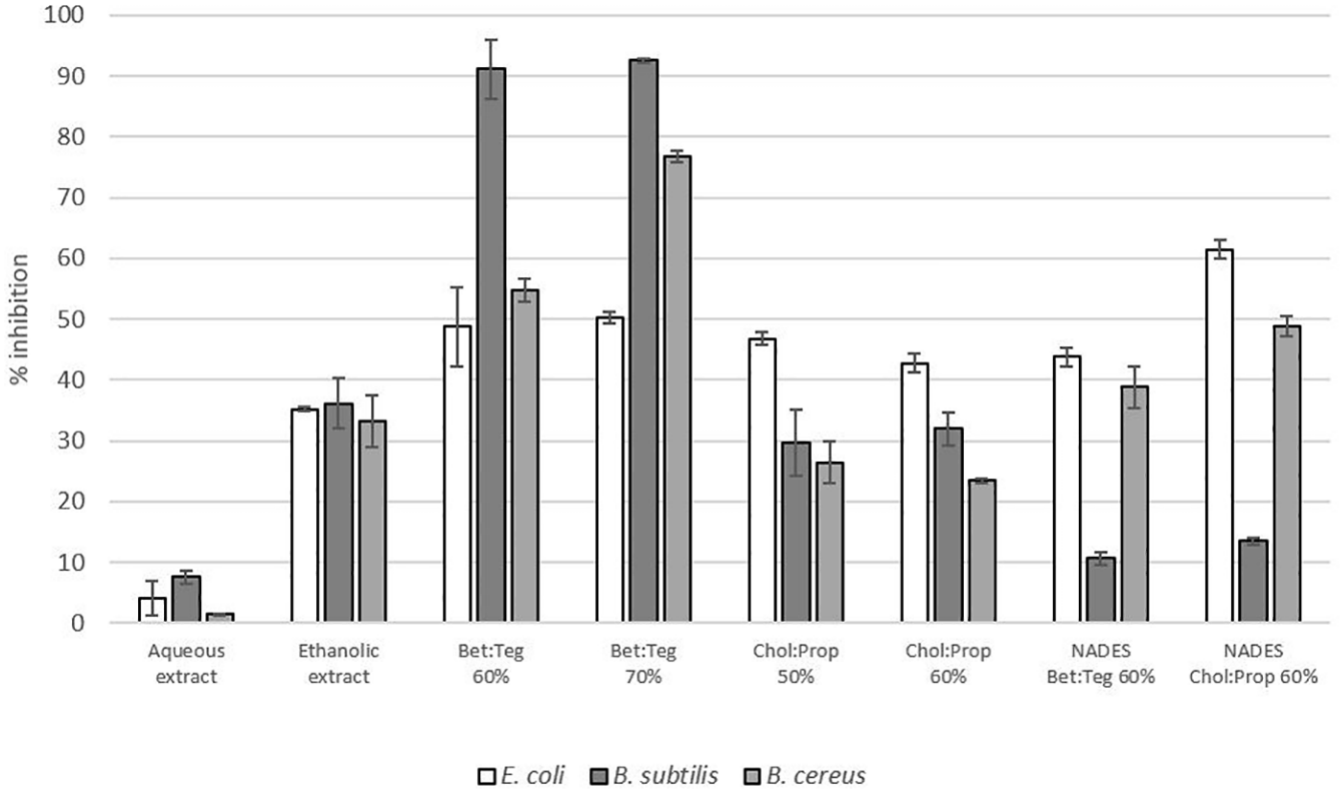
Inhibitory activity (%) of the extracts at the highest dilution and the corresponding solvents alone at the same dilution for Chol : Prop 60% and Bet : TEG 60%; the total polyphenol concentration (GAE mg/ml) in each extract was as follows: 830 (aqueous), 838 (ethanolic), 153 (Chol : Prop 50%), 141 (Chol : Prop 60%), 101 (Bet : TEG 70%), and 148 (Bet : TEG 60%).

In order to assess if there is any antimicrobial synergistic effect of NADES solvents, the antimicrobial activity (% inhibition) of extracts at the highest dilution and the corresponding solvent solutions for Chol : Prop 60% and Bet : Teg 60% are compared in **Figure 5**. In the case of Chol : Prop, the antimicrobial activity against *E. coli* and *B. cereus* is higher for the solvent alone than for the extract while the extract has higher activity against *B. subtilis* than the solvent alone, suggesting a synergistic effect (all differences statistically significant at *p* < 0.05). Propylene glycol has previously been reported to have antimicrobial activity against *S. aureus* and *E. coli* (Kinnunen and Koskela, 1991); hence, most probably, the high activity found for Chol : Prop against *E. coli* and *B. cereus* can be ascribed to this component. Similarly, Wojeicchowski et al. (2021) found that Chol : Prop 50% inhibited the growth of *E.coli*, *Clostridium perfringens*, *Listeria monocytogenes*, and *Salmonella* spp. by the disk diffusion sensitivity method. For Bet : Teg, the activity of the extract was higher than that of the solvent alone against *B. cereus* and particularly against *B. subtilis*, suggesting a synergistic effect (all differences were statistically significant at *p* < 0.05). Overall, these results show that the highest antimicrobial activity was obtained with the Bet : Teg extracts, even higher than with the ethanolic extract.

On the other hand, the inhibitory effect of aqueous and ethanolic extracts was due solely to the components of the extract, possibly the polyphenols, as the samples were freeze dried and resuspended in water, eliminating the effect of the solvent (ethanol). Interestingly, the ethanolic extract had a lower MIC and a higher inhibitory activity than the aqueous extract for the three tested bacteria, which suggests differences in phenolics profile between the extracts. As ethanol is less polar than water, the ethanolic extract could contain more hydrophobic polyphenols that seem to have a stronger inhibitory effect for the tested bacteria.

## 5 Conclusion

This work proves that NADES, in particular Chol : Prop, have demonstrated to be as effective as other conventional green solvents in the extraction of polyphenols from SCG, with the added advantage of NADES extractions being carried out at milder temperature conditions, without flammable solvents, and using sustainable and natural compounds. Further optimization of NADES extraction could result in an advantageous process for industrial applications as it avoids the use of flammable solvents and could result in energy savings by operating at milder temperature conditions than the other green solvents. Moreover, Chol : Prop showed a similar selectivity of extraction to water; their extracts were enriched in polyphenols as well as in protein while Bet : Teg and ethanol extracts had very similar composition with higher selectivity for reducing sugars than the water and Chol : Prop extracts. The differences in selectivity between the two NADES clearly showed that differences in extraction were mainly due to differences in chemical interactions with the SCG components; viscosity had an effect on Bet : Teg at different dilutions where reduced viscosity led to higher extraction yield. Overall, Chol : Prop was found to be the best extractant (with little difference between 50% and 60%) in terms of extraction yield of polyphenols, caffeine, and selectivity. In terms of antioxidant activity, Bet : Teg was superior to Chol : Prop and similar to ethanol. Regarding antimicrobial activity, both NADES led to a 10-fold reduction in MIC partly due to the NADES components. Yet, the higher antimicrobial activity found for the NADES extracts could be of interest when producing an extract with multiple functionalities of relevance for a food ingredient, including antioxidant and antimicrobial activity. Thus, further purification of the polyphenols may not be necessary.

## Data availability statement

The raw data supporting the conclusions of this article will be made available by the authors, without undue reservation.

## Author contributions

Data curation, formal analysis, methodology, and writing—original draft: AG-R. Methodology and formal analysis: LP. Conceptualization: Paula Jauregi. Funding acquisition and resources: PJ. Writing—review and editing: PJ. Supervision: PJ. All authors contributed to the article and approved the submitted version.
