# Supplementary material for: Natural deep eutectic solvents as a green extraction of polyphenols from spent coffee ground with enhanced bioactivities
Source: Front Plant Sci. 2023 Jan 11;13:1072592. doi: 10.3389/fpls.2022.1072592 (PMC9874221; doi:10.3389/fpls.2022.1072592)
Supplement: Supplementary file 1 [file DataSheet_1.pdf]

Supplementary Material

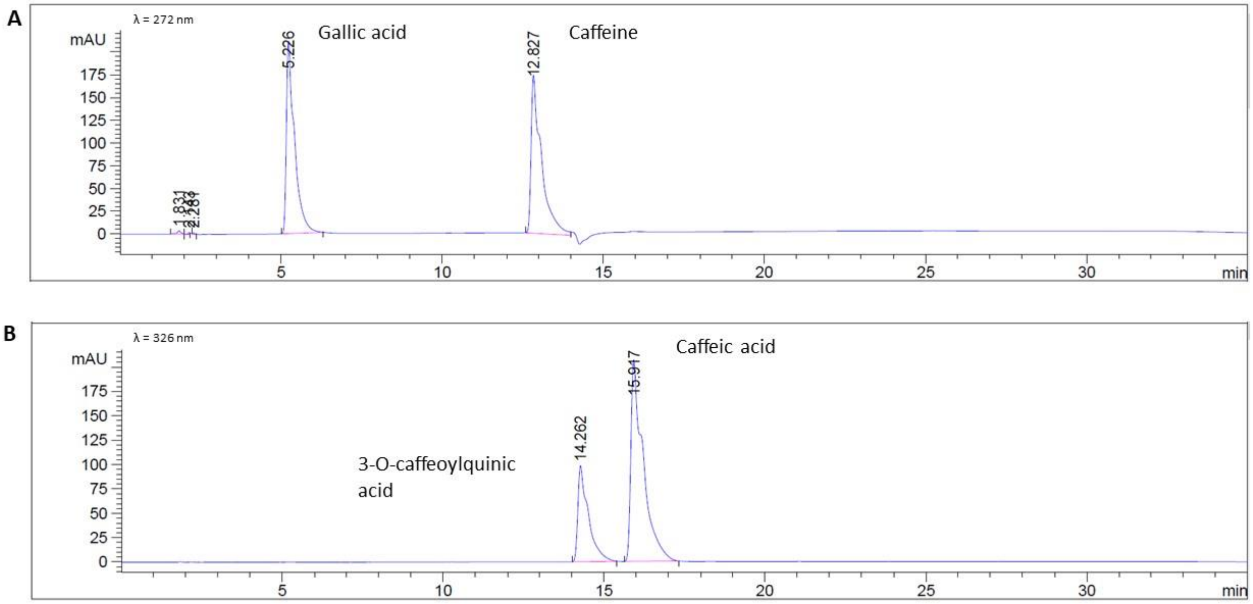

Supplementary Figure 1. Chromatograms of the standards and peak identification at (A) λ = 272 nm and (B) λ = 326 nm.

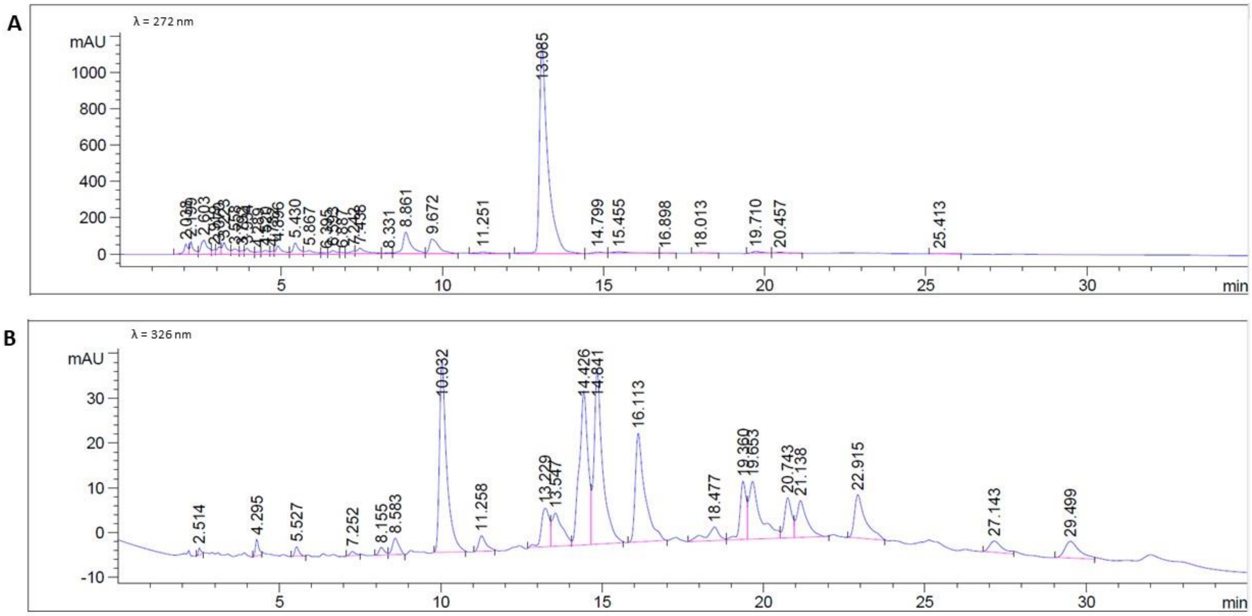

Supplementary Figure 2. Chromatograms of the aqueous extract at (A) λ = 272 nm and (B) λ = 326 nm.

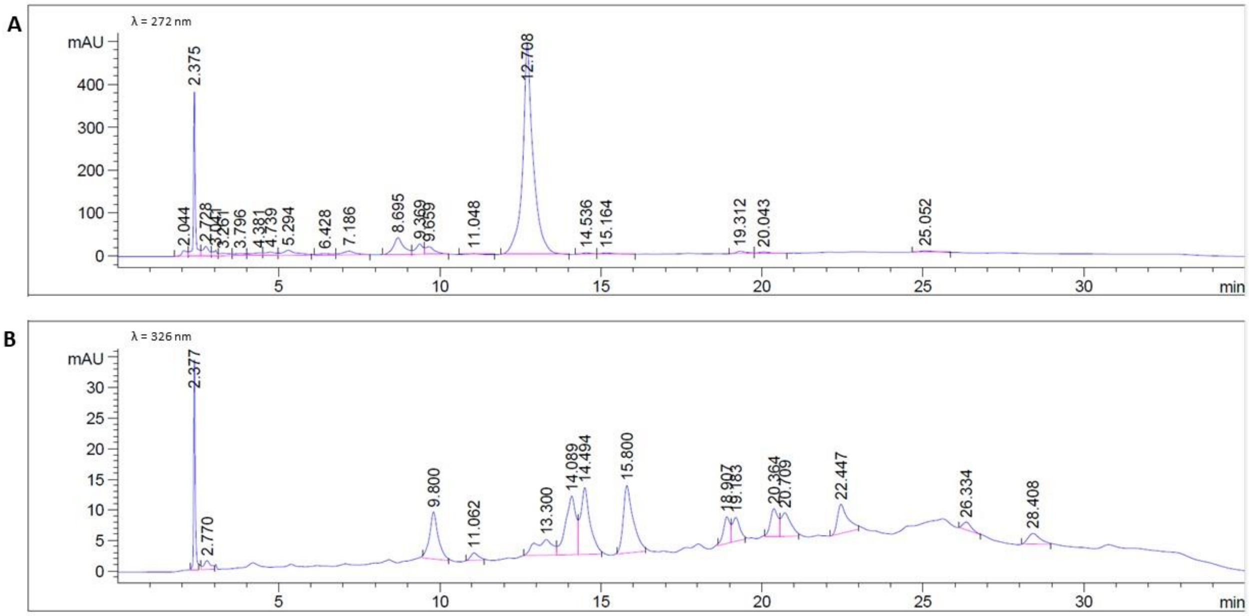

Supplementary Figure 3. Chromatograms of the ethanolic extract at (A) λ = 272 nm and (B) λ = 326 nm.

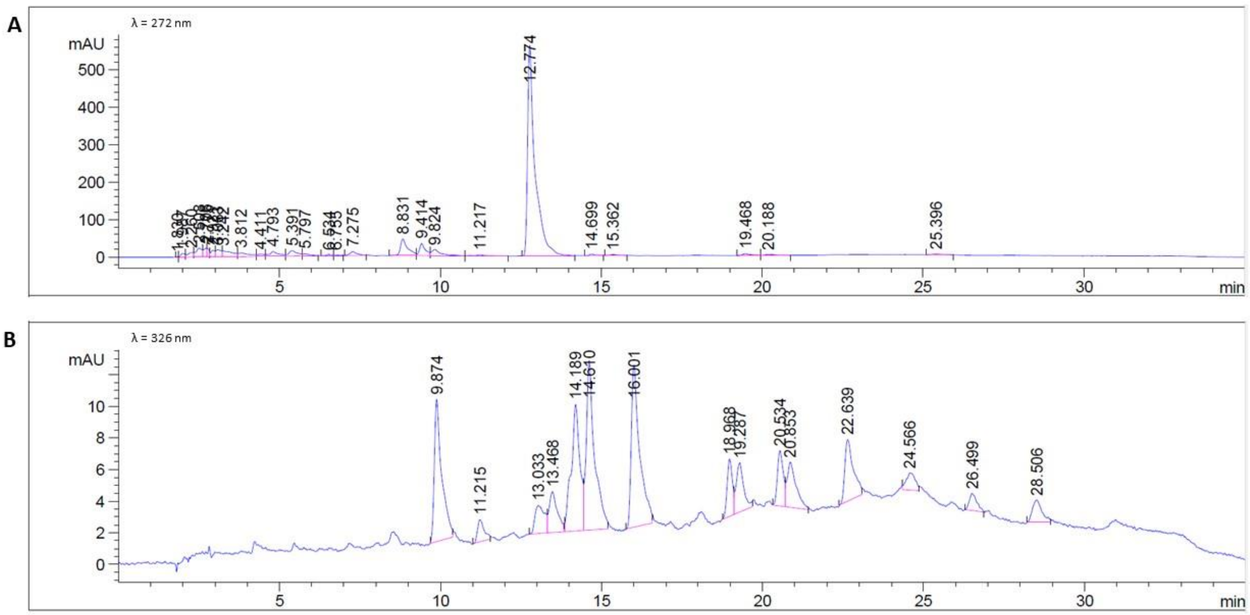

Supplementary Figure 4. Chromatograms of the Bet:Teg 60% extract at (A) λ = 272 nm and (B) λ = 326 nm.

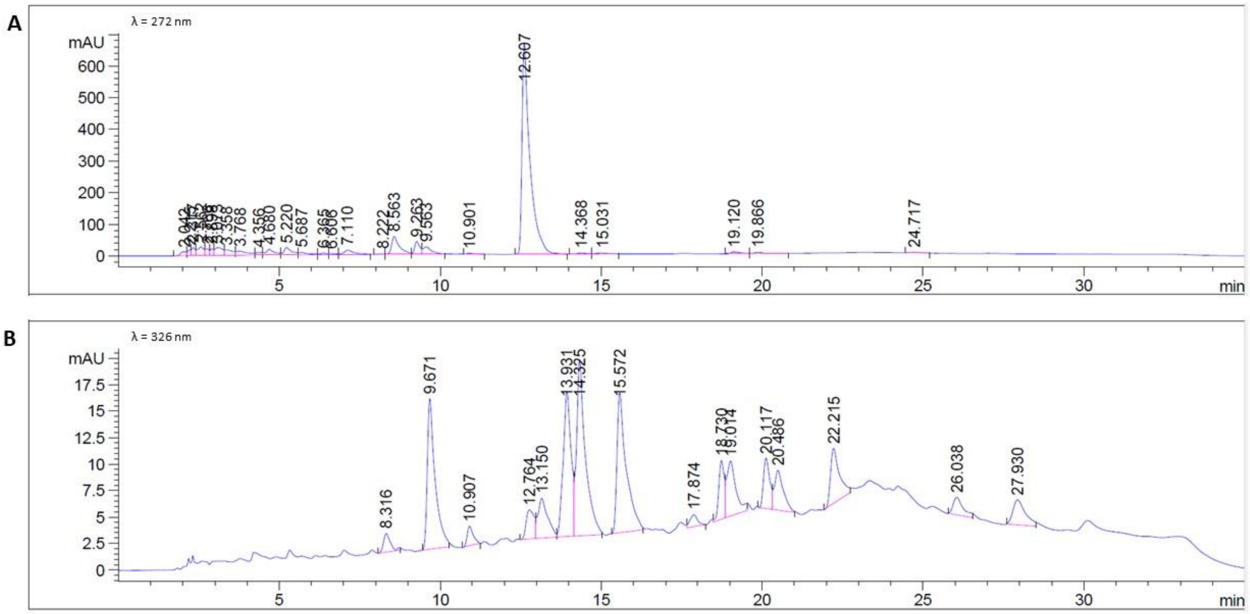

Supplementary Figure 5. Chromatograms of the Chol:Prop 50% extract at (A) λ = 272 nm and (B) λ = 326 nm.
